# A potential novel technique for measurement of pulp volume on periapical radiography: A pilot study

**DOI:** 10.1002/kjm2.12814

**Published:** 2024-03-06

**Authors:** Samed Şatır, Şelale Özel, Kaan Orhan

**Affiliations:** ^1^ Department of Oral and Maxillofacial Radiology, Faculty of Dentistry Karamanoglu Mehmetbey University Karaman Turkey; ^2^ Department of Oral and Maxillofacial Radiology, Faculty of Dentistry Altınbaş University Istanbul Turkey; ^3^ Department of Dentomaxillofacial Radiology, Faculty of Dentistry Ankara University Ankara Turkey; ^4^ Ankara University Medical Design Application and Research Center (MEDITAM) Ankara Turkey; ^5^ Department of Oral Diagnostics, Faculty of Dentistry Semmelweis University Budapest Hungary

**Keywords:** CBCT, ImageJ, periapical radiography, pulp volume

## Abstract

Pulp volume can be assessed during dental treatment. Three‐dimensional imaging techniques are not routinely used for this purpose because of high radiation doses. This study aimed to develop a novel method to measure pulp volume using periapical radiography. In this study, cone‐beam computed tomography (CBCT) was used as a reference method. Periapical radiography and CBCTs obtained from the same patients (*n* = 32) were recorded. Pulp volume was determined by observing the density differences between the pulp and peripheral structures using ImageJ. A method of graph and volume calculation was developed for each tooth. The Shapiro–Wilk test and Mann–Whitney *U* test were used to show normality and non‐normal distributions. The Bland–Altman plot was used to show the scattering of the mean versus difference values of the measurements of the two methods used to calculate the pulp volume. Normality was evaluated using the Shapiro–Wilk test. CBCT measurements are normally distributed (*p* = 0.307), while ImageJ is not normally distributed (*p* = 0.027). Therefore, the mean difference between the two groups was analyzed using the nonparametric Mann–Whitney *U* test. There was a statistically significant difference between the CBCT and ImageJ measurements (*p* = 0.01). According to Spearman's correlation analysis, the results obtained from the novel method were moderately correlated with those obtained from the reference method (*r* = 0.444). The results of this study indicated that a novel method‐based Java software can be used to calculate pulp volume using low‐dose radiation containing periapical radiography.

## 1 INTRODUCTION

The pulp chamber becomes narrow because of the production of secondary or tertiary dentin with aging or infection. Therefore, pulp volume is a clear marker for dental age estimation, evaluation of root canal morphology, diagnosis of canal necrosis, and identification of caries‐related pulp. In cases where the clinical presentation of pathological components, such as trauma and caries, affects the pulp, pulp volume is an important indicator, especially for the treatment areas of dentistry. Healthy teeth that do not have a pathological history can be used for age estimation in forensic medicine.^1^, ^2^

Periapical radiographs are not suitable for evaluating the pretreatment pulp cavity or endodontic treatment effect on the pulp cavity because superimposing the shadows of the structures causes misinterpretation of the periapical radiograph.^3^ Cone‐beam computed tomography (CBCT), a three‐dimensional (3D) radiographic technique, is the most suitable method for evaluating pulp volume.^2^, ^4^, ^5^ However, reasons such as high radiation dose, cost, and time loss limit the use of CBCT.^3^, ^5^ In addition, the calculation of a sensitive and relatively small area, such as the pulp volume with CBCT, by a dentist or radiologist increases the possibility of human error and causes the results to be subjective. These disadvantages in obtaining objective data may cause difficulties in adapting artificial intelligence (AI) applications, which are gaining momentum in every field of dentistry, for pulp volume calculation.^6^

ImageJ (Wayne Rasband, National Institutes of Health‐NIH, Maryland, USA) is an image processing program that can perform a variety of tasks, such as evaluating the trabecular structure, density histogram, and automatic cell counting.^7^, ^8^, ^9^ Histogram analysis shows the distribution of gray values in the radiographic image. The discrepancy in the gray values of different tissues can be measured using this feature of ImageJ.

For pulp volume calculation, the preference for methods with high radiation safety, such as periapical radiography, depends on the accuracy of the data obtained with these methods. Therefore, this study aimed to convert density differences between dental pulp and surrounding tissues in periapical radiography into numerical data using ImageJ software and to show its effectiveness in measuring pulp volume.

## 2 MATERIALS AND METHODS

### 2.1 Ethics

This research was conducted in accordance with the Helsinki Declaration. Ethical approval for this retrospective study was obtained from the Altınbaş University Clinical Research Ethics Committee (2021/92).

### 2.2 Study design

Gray values in the images reflect the thickness and density of the tissues.^9^ Dental pulp casts a dark area called radiolucent on the radiograph because of a more attenuated x‐ray beam. Calcification in the dental pulp can be considered when the difference in density between the dentin and the pulp decreases.^3^ An aggregate of gray values in the dental pulp can reflect pulp volume. The Histogram tool in ImageJ allows the conversion of the density of the tissues to gray values as numeric data.

In this study, pulp volume was calculated using Mimics software, and outcomes were compared with the findings of the novel model using ImageJ.

### 2.3 Sample selection

Single‐canaled teeth were included in this study to impede the superimposition of the dental pulp. The CBCT images confirmed the root canal morphology of the selected teeth. Initially, 223 teeth with periapical radiographs and CBCT images were determined. The Exclusion criteria were inadequate image quality (30 teeth), multicanaled teeth (17 teeth), teeth that had undergone endodontic treatment (3 teeth), teeth with open apexes (2 teeth), teeth with caries (55 teeth), teeth that had a trauma history (8 teeth), teeth with a periapical lesion (74 teeth), and root canals with internal resorption (2 teeth). After assessing the radiographic images according to the exclusion and inclusion criteria, we identified 32 teeth to measure pulp volume.

### 2.4 Pulp‐volume measurement on periapical radiography: The novel method

All periapical radiographs using parallel projection were performed with the Gendex Expert DC system (Gendex Dental Systems, Hatfield, USA) with exposure parameters of 65 kVp, 7 mA, 0.05 sn, and 65 kV, 7 mA, and 0.08 sn from the incisor and premolar teeth, respectively.

**FIGURE 1:**
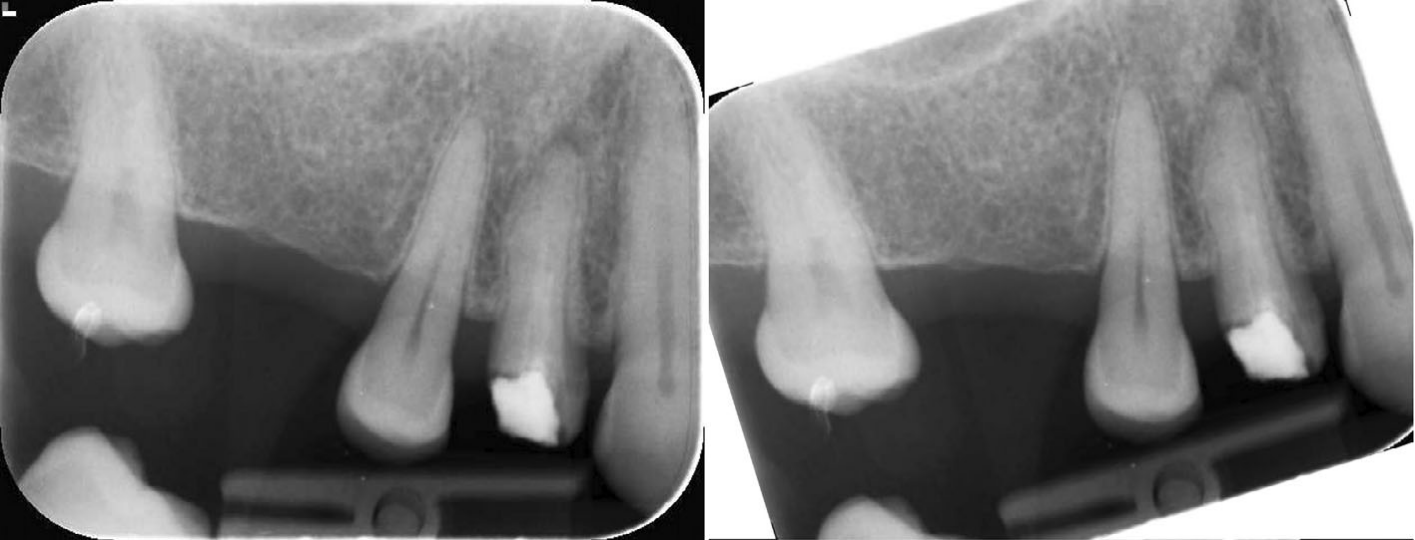
Vertical positioning of the periapical radiograph (second premolar) to be analyzed with Paint 3D.

**FIGURE 2:**
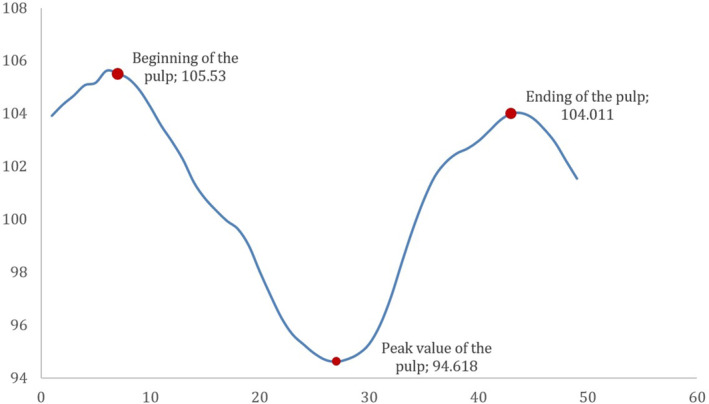
Graphic example showing the Mean values of the teeth according to the mesiodistal direction.

**FIGURE 3:**
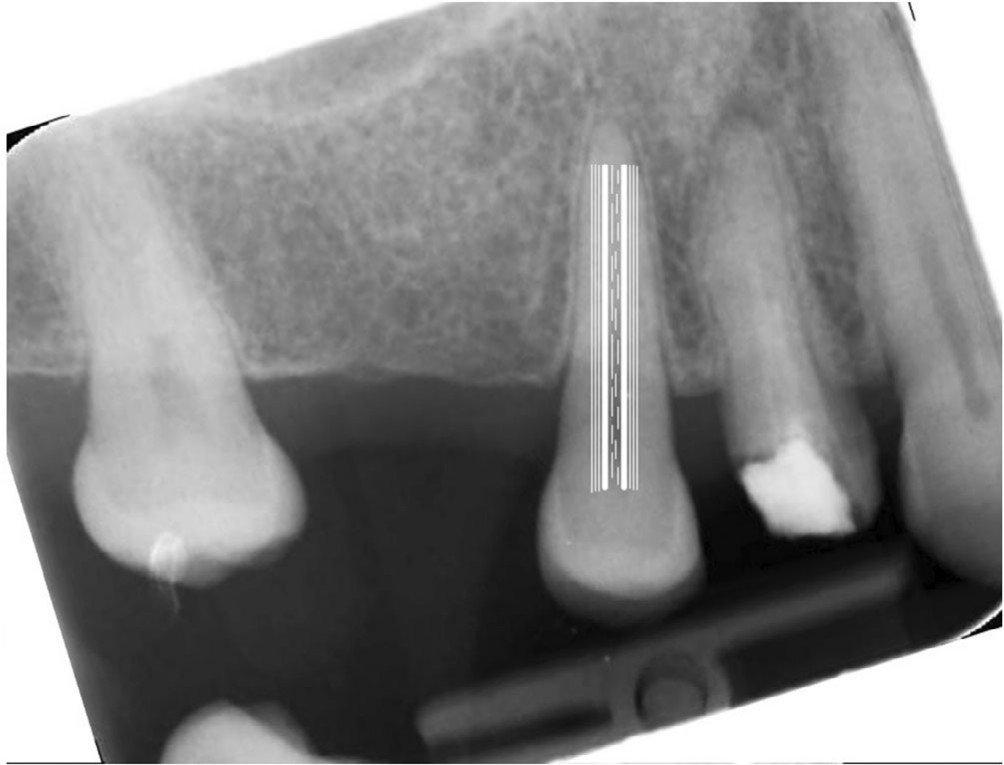
Illustration of columns analyzed with ImageJ in dentin (thin white line), pulp–dentin border (bold white line), and pulp (dashed line). Each column is of equal size in analysis. They are represented in different sizes to make it clear in the illustration.

**FIGURE 4:**
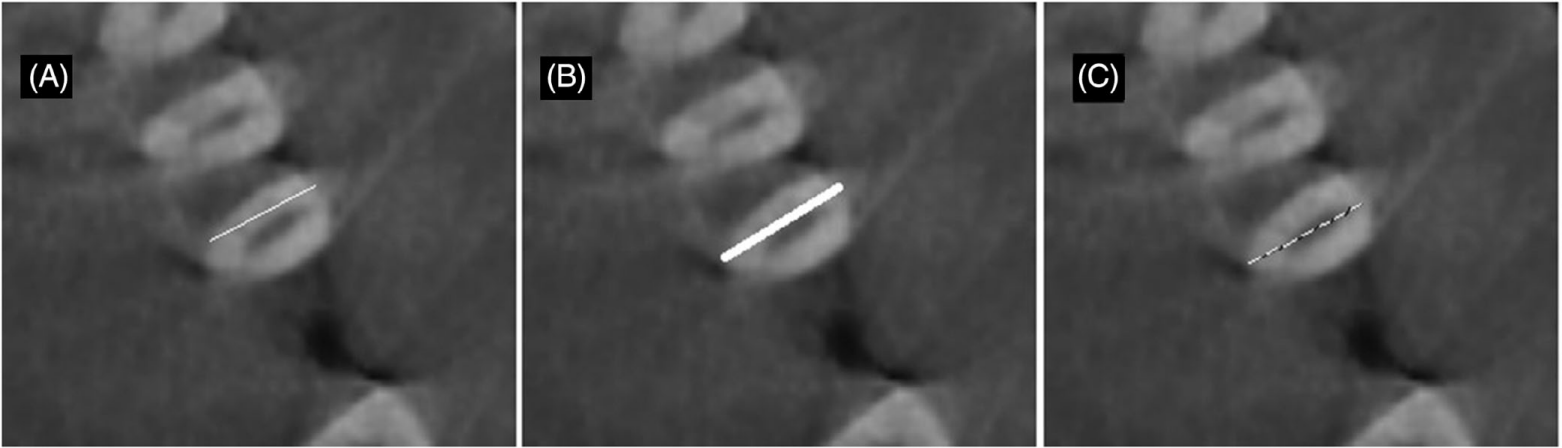
Cone‐beam computed tomography axial section illustration showing the variation of Mean value with dentin (thin white line‐A), pulp–dentin border (bold white line‐B), and pulp (dashed line‐C) in columns analyzed with ImageJ.

**FIGURE 5:**
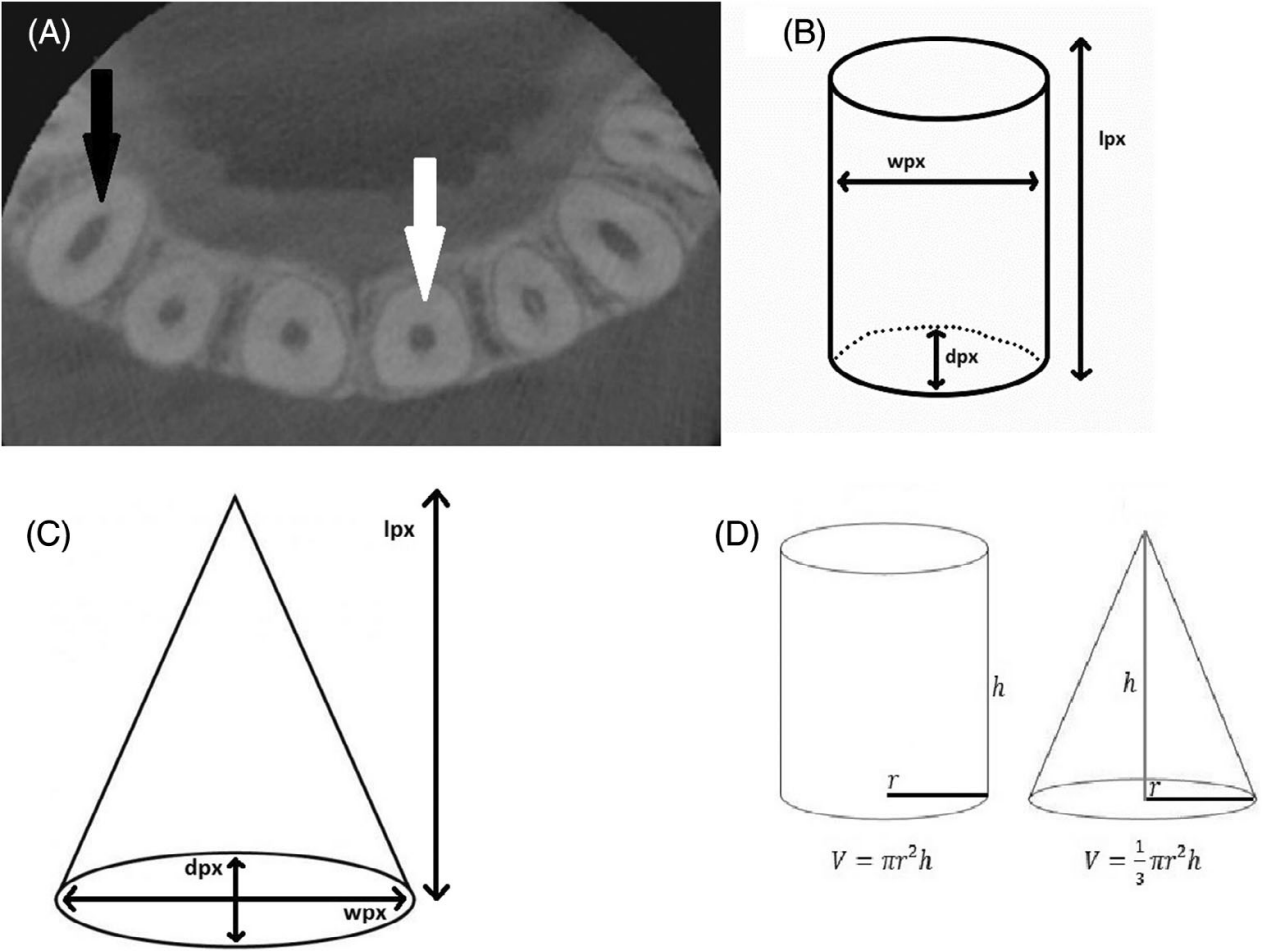
(A) Elliptical expansion in the buccolingual direction (black arrow) or oval (white arrow) shape of the pulp cavity in anterior teeth in the CBCT axial section (B) Representation of the obtained lpx, wpx, and dpx values and the method of calculating the pulp volume on the geometric cylinder (C) Representation of the obtained pulp length (lpx), pulp width (wpx), and mean depth (dpx) values and the method of calculating the pulp volume on the geometric cone (D) Mathematical volume formulas of geometric shapes that can be used to calculate pulp volume.

ImageJ software was used for pulp‐volume measurements. Periapical radiography was converted into TIFF format. To take the teeth in the vertical position, radiography was rotated on Paint 3D (Microsoft, Washington, USA; Figure 1). The image was calibrated for the 30 mm× 40 mm dimension of the phosphor plate. Columns with a width of one pixel and a length equal to the length of the dental pulp were determined. Using the ImageJ/Analyze/Histogram command, the mean width values for each column along the mesiodistal dimension (Mean) and the length of the teeth (Count) were recorded on Windows Excel (Microsoft, Washington, USA). According to the calculations for each tooth, the trend line in the graph usually climbed up to the pulp border and then fell gradually. A peak of the trend line, the lowest value, usually shown near the center of the pulp, then rising to the pulp border was observed (Figure 2). The spectrum of the values through the mesiodistal dimension of the teeth allows the identification of the maximum and minimum dentin and pulp densities (Figure 3). A reason for this pattern is that as dentin thickness in the buccolingual direction increased, the trend line rose from the tooth border to the pulp border, and the decline in the trend line from the pulp border to the center was explained by the enlargement of the pulp (Figure 4). The peak of the pulp border on both sides of the graph was determined to be the beginning of the pulp, and the peak value in the middle of the pulp was identified as the widest area of the pulp. Each value, from the distal pulp border to the distal peak point, was subtracted. The same procedure was applied to the mesial side. The numerical data obtained from the subtraction process was added. Each column's subtraction result, included in the pulp, was collected. To convert pixels to mm, the number of pixels covering the pulp length (lpx) and pulp mesiodistal width (wpx) was used on the number 2 phosphor plate (30 mm × 40 mm). To convert the pulp depth to mm, the pixel equivalent of the numerical value obtained using ImageJ was determined first. This was based on the knowledge that the pulp cavity shape of the anterior teeth in the axial section was oval or elliptical, expanding in the buccolingual direction (Figure 5A). Because the degree of elliptical shape in the pulp cavity cannot be standardized, it was accepted that all teeth included in the study were oval in every axial section of the pulp, and the thesis was accepted that the width in the mesiodistal direction and the depth in the buccolingual direction would have the same value. According to this thesis, a coefficient was obtained that would match the mean depth value (dpx) obtained from all teeth and the mean mesiodistal width value. The height, width, and depth of the pulp were determined as lpx, wpx, and dpx in pixel units, respectively. However, the volume calculation using these values was similar to calculating the volume of a cylinder (Figure 5B). Considering that the pulp resembles a cone narrowing from coronal to apical rather than the cylinder (Figure 5C), the volume calculated with lpx, wpx, and dpx values should be reduced by ~67% (Figure 5D).

### 2.5 Pulp‐volume measurement on CBCT images/reference method

All CBCT scans were performed with NewTom Vgi Evo (QR, Verona, Italy) with a voxel size of 0.3 mm using NNT Software. Tomographic images were converted into the DICOM format using the NNT viewer software (Version 2.21). To calculate pulp volume, the MIMICS software (Version 10.01; Materialise N.V., Belgium) was used. A new mask was created and edited to separate the teeth and pulp from the surrounding anatomical structures. For the segmentation procedure, the threshold was set on the teeth. The threshold feature segments the object according to the density value within the user‐selected threshold range. After applying the threshold, to eliminate, scatter, and disconnection pulp from the dentin, the “Multiple Slice Editing” tool was used. The “Multiple Slice Editing” tool allows manual editing of each slice.

All measurement and analysis stages of the reference and the novel methods were performed together by two observers (SS and SO).

### 2.6 Statistical analysis

The Shapiro–Wilk test and Mann–Whitney *U* test were used to show normality and non‐normal distributions. The Bland–Altman plot was used to show the scattering of the mean versus difference values of the measurements of the two methods used to calculate the pulp volume.^10^ The Spearman correlation test was used to determine the correlation between the two measurement methods.

## 3 RESULTS

Descriptive data about the age and gender of the patients whose pulp volume was measured and the pulp volume values measured by two different methods (CBCT and ImageJ) are given in Table 1.

**TABLE 1:** Descriptive statistics of the samples and pulp volumes.

	Female	Male	Total
*N* (min–max)	18	14	32
Age (min–max)	31.05 (19–52)	33.92 (25–60)	32.31 (19–60)
CBCT pulp volume in mm^3^ (min–max)	28.87 (11.01–39.66)	28.90 (8.84–50.1)	28.89 (8.84–50.1)
ImageJ pulp volume in mm^3^ (min–max)	20.37 (7.12–31.03)	25.39 (6.97–52.32)	22.57 (6.97–52.32)

Abbreviation: CBCT, cone‐beam computed tomography.

Normality was evaluated using the Shapiro–Wilk test. CBCT measurements are normally distributed (*p* = 0.307), while ImageJ is not normally distributed (*p* = 0.027). Therefore, the mean difference between the two groups was analyzed using the nonparametric Mann–Whitney U test. There was a statistically significant difference between the CBCT and ImageJ measurements (*p* = 0.01).

**FIGURE 6:**
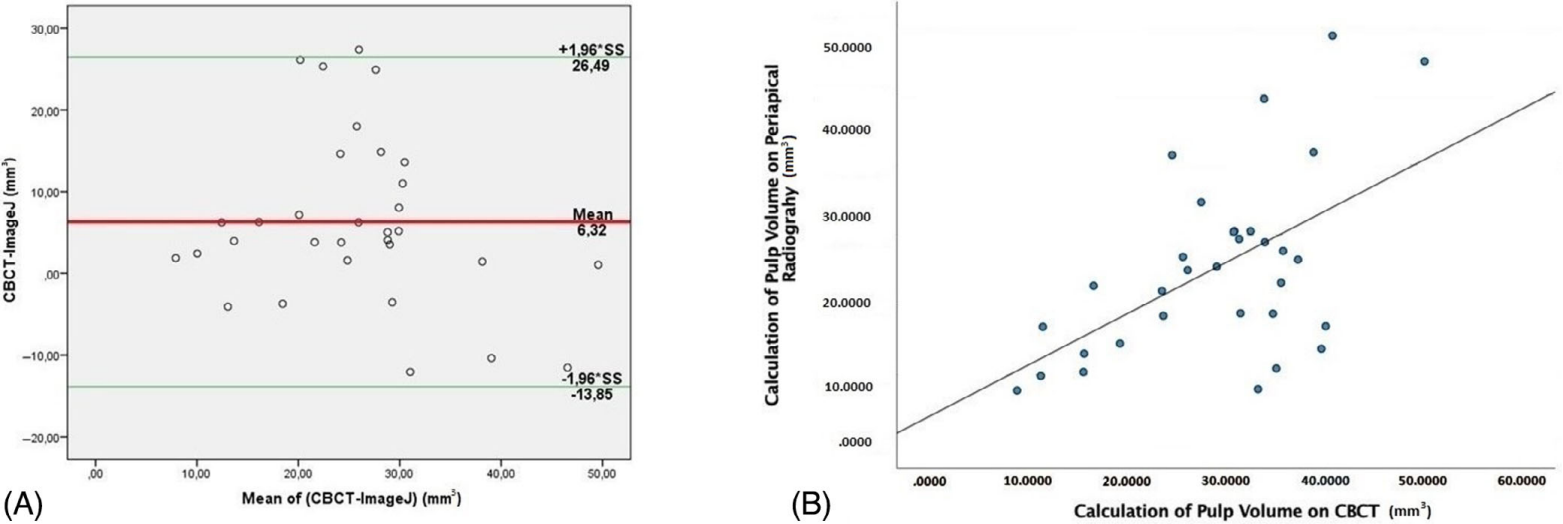
(A) Bland–Altman plot for cone‐beam computed tomography (CBCT) versus ImageJ measurement differences (green lines indicate 95% concordance limits). (B) Cone‐beam computed tomography axial section illustration showing the variation of Mean value with dentin thickness (dentin (thin white line), pulp–dentin border (bold white line), and pulp (dashed line)in columns analyzed with ImageJ.

On the *x*‐axis of the Bland–Altman graphs, the means for the measurement pairs, and on the *y*‐axis, the differences between the findings of the two methods are located (Figure 6A).^10^ According to the Bland–Altman plot results in the figure, the value obtained when the measurement is made with ImageJ may be 13.85 mm lower and 26.49 mm higher than the value obtained with CBCT. The differences between the measurement values of these two methods are not randomly distributed around zero (do not show a systematic distribution), so there is no good agreement between the two methods examined according to the Bland–Altman method (Table 2).

**TABLE 2:** Statistical results of the Bland–Altman method.

*N*	Valid	32
	Missing	0
Mean		6.3191
Median		4.5542
Std. deviation		10.29257
Range		39.43
Minimum		−12.07
Maximum		27.37

According to the Spearman correlation test, there is a moderately positive correlation between the two measurements (*r* = 0.444; Figure 6B).

## 4 DISCUSSION

Volume calculations using CBCT and micro‐CT depend on dentin–pulp borders. This novel method brings a different perspective to volume measurements with periapical radiography. Differences between the density of the intrapulpal and peripheral tissues (dentin, enamel, and alveolar bone) are the main determinants of how pulp volume is measured for this novel method.

In this study, pulp volume on periapical radiography was calculated on the basis of the distribution of the gray values of the periphery to the center pulp using ImageJ. Based on the research results, the novel method was found to be partially successful in calculating pulp volume. Despite micro‐CT being recognized as the reference standard for volumetric measurements, previous studies have indicated that CBCT also meets the standard for volumetric calculations in the maxillofacial area.^11^, ^12^, ^13^ Therefore, CBCT was accepted as a reference standard to evaluate the accuracy of this novel method of periapical radiography. Instead of Micro‐CT, CBCT is an essential technique to evaluate the effect of the hard and soft tissue structures on periapical radiography. Micro‐CT can be performed to obtain more reliable results; however, there are important limitations to this method, including the absence of evaluation of the superposition of the hard and soft tissue structures on the pulp because of the requirement of tooth extraction.^4^, ^14^

The pulp volume calculated using the new method was found to be lower than that of the CBCT measurements. Many technical reasons may have contributed to this difference, such as the fact that periapical radiographs and CBCTs were not taken on the same day, the dependence of the CBCT measurements on the person performing them, and the presence of positioning errors or density differences in the periapical radiographs. However, considering the study methodology, the assumption that all samples have an oval pulp in the axial section can be considered the main factor in the lower calculation of pulp volume with the new method compared with CBCT. Second, it can be shown that the principles of the new technique are based on the physiological characteristics of the pulp rather than the morphological features. While calculating the pulp volume with CBCT is based on the walls of the pulp chamber, the new method in this study is based on the density of the pulp chamber. Although the clinical history of the samples included in this retrospective study is not known, reasons such as the pulp containing vascular nerve packages or pulp calcifications may have caused the pulp to be calculated with a lower volume using the new method. Therefore, the new method can be used in various clinical procedures, namely in endodontic treatment or orthodontics, to identify pulpal calcifications or obliterations more than in forensic medicine.

The pulp volumes obtained using CBCT are consistent with the average pulp volumes reported in the literature.^15^, ^16^ The pulp volumes of the canine and maxillary anterior teeth were measured to be larger than those of the other samples. The pulp volumes of the mandibular anterior teeth were also calculated to be relatively small. The difference between the oval morphologies of the incisor and premolar dental pulps in the axial section may affect the pulp volume measured by CBCT and the pulp volume calculation with periapical radiography.

In a study using CBCT, the difficulty and subjectivity of determining the enamel volume using gray tones in the sections were mentioned.^4^ Likewise, the dentin–pulp border is determined by the clinicians to calculate pulp volume on CBCT. However, this leads to inaccurate results that are caused by human error. In this novel method, the assessment of the dentin–pulp border depends on the density of the tissues. The resulting numerical data are determined by the trend generated using ImageJ software, not the clinician. Differences in the numeric data through the mesiodistal dimension were used to determine the mesiodistal border of the pulp. Determination of the dentin–pulp border through the apical–coronal dimension by the clinician does not cause a significant deviation in the results. When the dentin–pulp boundary is determined to be longer in the coronal direction, the values (Mean) obtained from ImageJ decrease. However, the margin of error will decrease because the length of the pulp (Count) will increase. The possibility of obtaining objective data independent of human influence may facilitate the adaptation of AI applications related to pulp volume calculation, which has recently gained momentum in endodontics, orthodontics, and forensic dentistry.

In a study in which Magnetic resonance imaging (MRI) was used to calculate pulp volume, it was stated that it could be an alternative to CBCT as a radiation‐free method. In addition, it has been shown as another advantage that caries that could not be detected before MRI in a few extracted tooth samples can be diagnosed by MRI.^2^ However, because MRI is unsuccessful in imaging water‐free hard tissues such as bones and teeth compared with dental radiographs, the pulp volume calculation method with periapical radiography presented in this study may be an alternative in dentistry.^3^

**FIGURE 7:**
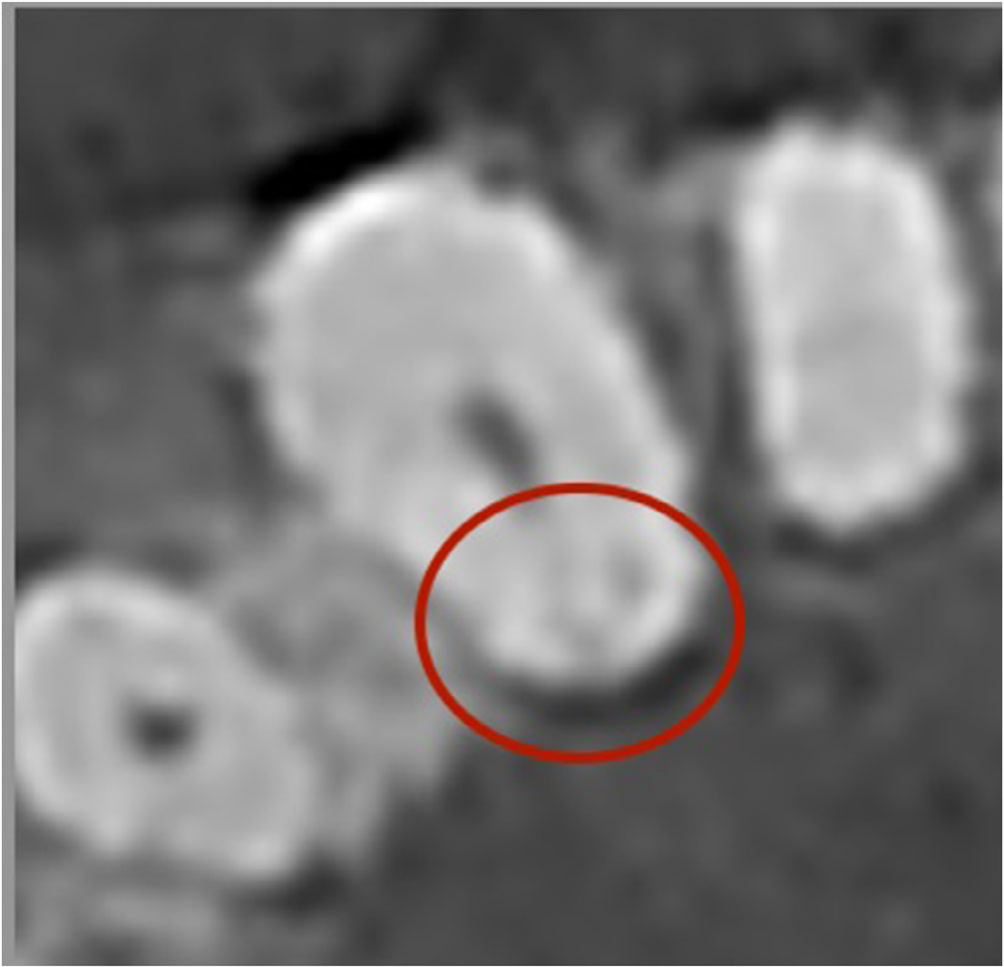
Cone‐beam computed tomography axial image of fractured tooth presenting graphical irregularity.

The graphics created using ImageJ in the cases of four patients in this study were found to be incoherent with those of the remaining patients. Fractures were found in the crown and root regions in an evaluation of the volumetric tomography images of one such patient, and the difference in density caused by these fractures may explain the irregularity of the slope of the graphic (Figure 7). When the dental volumetric tomography of another patient with an irregular graphic was evaluated, the mental foramen was found to be at the level of the apex of the tooth, and a partially depressed bone compared with the surrounding tissues was suggested to have resulted in a density difference in the root identified from periapical radiography.

One of the limitations of the study was that periapical radiographs and CBCTs from the same date were unavailable for some patients, given the retrospective nature of the study. Images were obtained 6 months, 1 year, and 2 years apart in 5, 6, and 1 patients, respectively, whereas the period between the periapical radiographs and CBCT was no longer than 1 month in the remaining 20 patients. Pulp volume decreases with age because of secondary dentin formation. A review of the literature revealed that pulp volume statistically significantly decreases by 1%–2.7% over 3–6 months.^17^, ^18^ Given that this study was designed as a pilot study, it was not deemed appropriate to obtain a second radiograph. The limited concordance between the two techniques may be because the radiographs were not taken simultaneously. Future studies would benefit from the evaluation of radiographs obtained on the same day, where possible.

Another limitation is that this method is only valid for single‐rooted/single‐canal anterior teeth. However, accessory canals are frequently encountered, even in teeth that are considered to be single‐rooted/canal. However, the presence of an accessory canal such as a root fracture, which was diagnosed due to a system error in the pulp volume calculation of a tooth in this study (Figure 7), may also make the tooth suspicious in unexpected situations that will occur in the volume calculation. The accessory canal can be diagnosed with additional radiographic imaging, such as CBCT.

The fact that the pulp volume calculated with the new method is in a wider range than that calculated with CBCT and the discrepancy in some samples may be related to the methodology. In this pilot study, a simple method was followed to support the thesis that pulp volume can be calculated from periapical radiography. Another limitation of this pilot study is that the number of samples was limited, and different intraoral digital radiography systems were not used. Pulp‐volume calculation with periapical radiography may become a technique with increased accuracy in new studies with larger sample numbers. Making the method more detailed and improving the coefficient used for mm^3^ conversion may allow both the improvement of pulp‐volume calculation and 3D modeling of the pulp.

## 5 CONCLUSION

Pulp volume is widely evaluated by clinicians in dentistry. In this study, a new method was developed using ImageJ software to calculate the pulp volume in periapical radiography. The results of this study indicated that there was a moderate correlation between the new and reference methods. New studies are needed to develop this method to calculate pulp volume with low‐dose radiation‐containing periapical radiography and to contribute to AI‐based dentistry applications.

Making the 3D model of the pulp using periapical radiography, a widely used radiographic technique, can be a useful aid in determining pulp volume with a low radiation dose and increasing the success rate of endodontic treatment.

## CONFLICT OF INTEREST STATEMENT

The authors declare that they have no competing interests.
